# Advances in Thermoelectric Composites Consisting of Conductive Polymers and Fillers with Different Architectures

**DOI:** 10.3390/molecules27206932

**Published:** 2022-10-16

**Authors:** Bingchen Huo, Cun-Yue Guo

**Affiliations:** 1 High & New Technology Research Center, Henan Academy of Sciences, Zhengzhou 450003, China; 2 School of Chemical Sciences, University of Chinese Academy of Sciences, Beijing 100049, China

**Keywords:** conductive polymer, thermoelectric, composites, architecture, carbon materials

## Abstract

Stretchable wireless power is in increasingly high demand in fields such as smart devices, flexible robots, and electronic skins. Thermoelectric devices are able to convert heat into electricity due to the Seebeck effect, making them promising candidates for wearable electronics. Therefore, high-performance conductive polymer-based composites are urgently required for flexible wearable thermoelectric devices for the utilization of low-grade thermal energy. In this review, mechanisms and optimization strategies for polymer-based thermoelectric composites containing fillers of different architectures will be introduced, and recent advances in the development of such thermoelectric composites containing 0- to 3-dimensional filler components will be presented and outlooked.

## 1. Introduction

**Figure 1:**
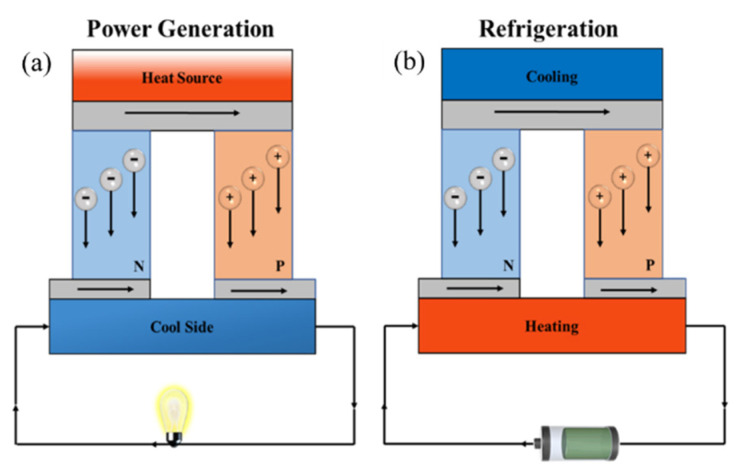
Schemes of TE devices. (**a**) Seebeck effect. (**b**) Peltier effect.

As a global strategy, carbon neutrality poses numerous questions regarding the development of clean energy, which has become the core of energy plans for most countries. As a form of green renewable energy, thermoelectricity has emerged [1,2,3,4]. Thermoelectric (TE) materials are able to interconvert between heat and electricity based on the Seebeck effect and Peltier effect (Figure 1), which has been found for over 100 years [5]. TE devices possess plenty of properties such as no noise, no vibration, no gaseous emission, widespread waste heat sources, etc., and can be evaluated by the figure of merit *ZT = S*^2^*σT/κ* (1), which is dimensionless, where *S* is Seebeck coefficient, *σ* is the electrical conductivity of materials, *T* is absolute temperature, and *κ* is the thermal conductivity [6,7]. Although TE materials are promising and have lots of advantages, predominant inorganic TE materials (such as Sb_2_Te_3_, Bi_2_Te_3_, etc.) are extremely limited in their application for wearable, portable, and implantable electronic devices and flexible robots due to the scarcity, toxicity, expensiveness, and difficulty in processing [8,9,10,11]. Fortunately, composites which consist of conductive polymers and fillers with different architectures, such as inorganic semiconductors or carbon nanoparticles, can balance properties with needs. They could not only approach the TE performance of inorganic materials to a certain extent but also have excellent processability similar to that of organic thermoelectric (OTE) materials [12]. Nowadays, the achievements of TE devices depend on the development of OTE materials. A perfect device should be constructed from high-performance TE materials [13]. Furthermore, a TE generator (TEG) generally consists of p-type and n-type TE couples alternately, in which an electric relay combines with a thermos relay in series. Though there are many research achievements in p-type semiconductors, there is no significant progress in n-type materials, which impedes the development of high-performance and air-stable TE modules.

In this review, conductive polymers and fillers with different architectures in TE composites are elaborated on. The *ZT* value plays a pivotal role in evaluating TE materials’ performance, and it depends on the values of *S*, *σ*, and *κ* in Formula (1). An ideal TE material needs a high Seebeck coefficient, high electrical conductivity, and low thermal conductivity simultaneously. However, it seems a paradox to trade-off between these interdependent parameters and improving one may inevitably hamper the others. We noticed that Petru et al. reviewed how nanoparticles influenced the thermal and electrical conductivity of composites [14]. This work could guide how to balance the *σ* and *κ* in the composites required. Constructing composite materials is one prospective solution to avoid this dilemma; this can be conducted by adding TE fillers (such as inorganic semiconductors or carbon nanoparticles) of high Seebeck coefficient and/or high electrical conductivity into the low thermal conductivity polymers. This strategy is not just a simple commixture of mixing every component, as the enhancement of TE performance needs extra carrier pathways and an energy-filtering effect with introduced interfaces [15,16,17]. To decrease the thermal conductivity of TE composites, different architecture fillers are employed in corresponding polymer systems to influence lattice spacing dimensions. In other words, those composite parameters would be contributed by their composition/morphology.

Recently, plenty of research progress on TE polymer composite has been realized, and these are optimal solutions to develop flexible TE devices. It is believed that material architectures work on the interfaces of constituents in TE models with different applied actions; thus, it is meaningful to summarize and prospect in this regard. As there have been few reviews on this topic before, this mini-review proposes to present a new perspective on the construction and application of polymer-based TE composites in addition to their latest advances. We expect that this review could shed light on an in-depth understanding of the design and development of novel conductive polymer-based high-performance TE composites.

## 2. Zero-Dimensional (0D) TE Materials

### 2.1. Zero-Dimensional (0D) Materials

Materials with three dimensions on the nanoscale or made up of them as basic units are often referred to as 0D materials, such as nanoparticles, quantum dots, fullerene, etc. The most significant difference between traditional semiconductor-based thermoelectric devices and micro TEs is the structure size. The distance traveled by an excited hot electron before it relaxes to thermal equilibrium under inelastic scattering is the relaxation length [1,18,19]. However, the relaxation length is usually only a few tens of nanometers at room temperature, much smaller than the size of conventional TE devices. In semiconductor TE devices, we can assume that each point of the system is in local thermal equilibrium, which can be described by Boltzmann transport theory [20,21]. When the size of micro and nano TE devices is close to or even smaller than the electronic relaxation length, the system becomes non-local, and Boltzmann’s theory is no longer applicable [22,23]. In addition, there will be a variety of quantum effects, such as the quantum interference effect in the system, which has greatly stimulated people’s great interest in using particle theory to study the TE effect of nanomaterials. 0D materials, due to their small structural size, can efficiently scatter phonons, thereby reducing lattice thermal conductivity and achieving higher *ZT* values [24,25].

#### 2.1.1. Fullerene

**Figure 2:**
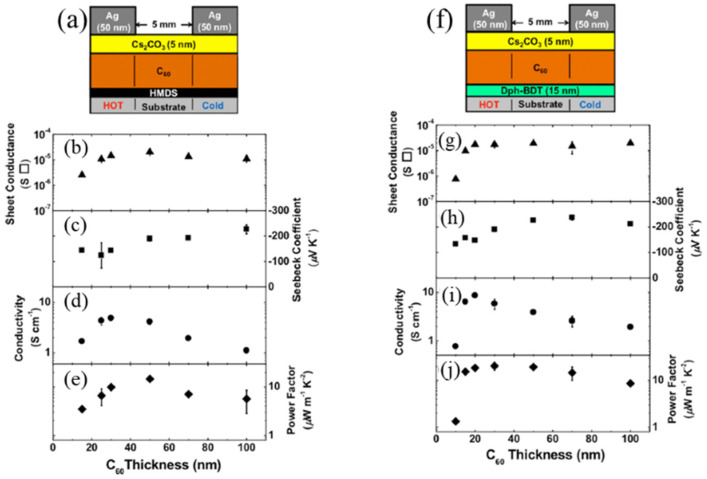
Schemes of C_60_/CS_2_CO_3_ bilayer samples. (**a**) Schematic view of the device of HMDS-modified substrates. (**b**–**e**) Sheet conductance, Seebeck coefficient, electrical conductivity, and power factor measured at *T* = 298 K of HMDS-modified substrates. (**f**) Schematic view of device of Dph-BDT layer. (**g**–**j**) Sheet conductance, Seebeck coefficient, electrical conductivity, and power factor of the device measured at *T* = 298 K of Dph-BDT. Adapted from ref. [29] with permission from AIP Publishing.

**Figure 3:**
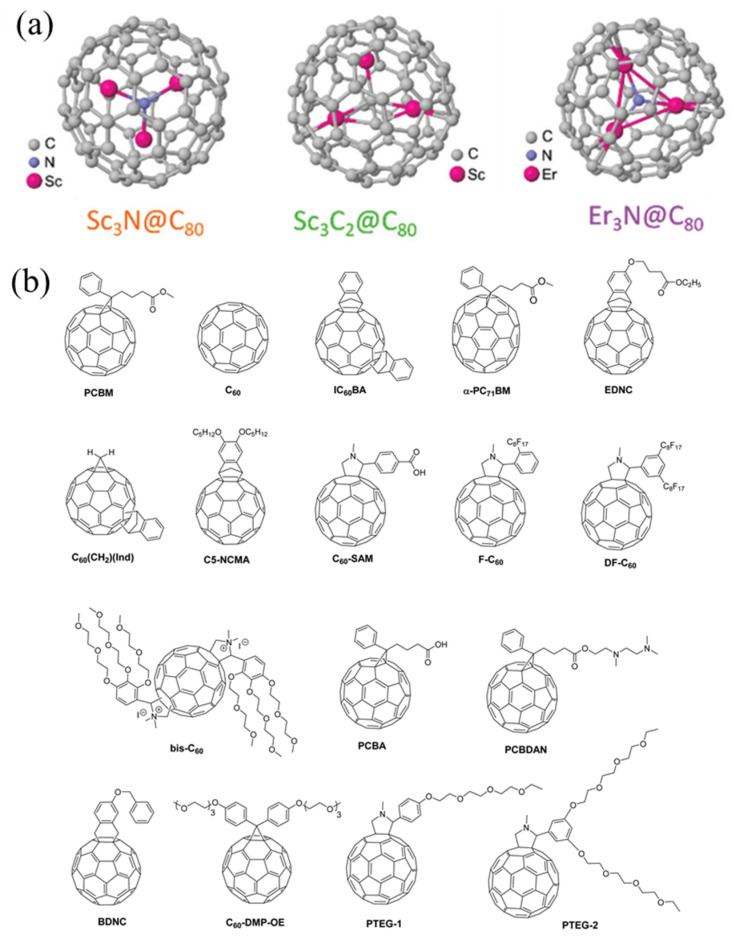
(**a**) Schematic of EMFs, Sc_3_N@C_80_, Sc_3_C_2_@C_80_, and Er_3_@C_80_. Adapted from ref. [30] with permission from the Royal Society of Chemistry. (**b**) Chemical structures of fullerene and fullerene derivatives. Reproduced from ref. [35] with permission from the Royal Society of Chemistry.

Fullerenes are widely studied for potential applications ranging from sensors and photovoltaic cells to nanostructured devices with their spherical geometry and unique optical and electronic properties. Recently, plenty of studies showed that C_60_ is a kind of robust TE material which has negative thermopower [26,27,28]. Therefore, C_60_ placed great expectations on developing n-type organic materials. Chihaya et al. utilized a bilayer structure composed of fullerene and Cs_2_CO_3_ to construct high-performance n-type TEs. The power factor of n-type TE elements reached 20.5 μW m^−1^ K^−2^ at room temperature [29] (Figure 2). Although fullerenes have already been applied to several devices, C_60_ cannot exhibit high power factors due to its relatively uniform charge distribution. Fullerene derivatives, such as endohedral metallofullerenes (EMFs) with highly homogeneous charge distribution, can introduce a high value of the Seebeck coefficient. For instance, EMFs (Figure 3a) have a higher Seebeck coefficient than C_60_, with both positive and negative signs of the Seebeck coefficient, implying bi-thermoelectric materials. The high-performance thermoelectricity of EMF roots in orientations corresponds to a high value [30]. To accommodate more fields, fullerene derivatives (some fullerene derivatives in Figure 3b) are required with new properties, such as strong polarity, electron-donating, and water solubility. The occurrence of soluble methanofullerene derivative (PCBM) is a breakthrough for low-cost solution processing of fullerene derivatives in electronics [31,32]. Wei et al. first doped PCBM with N-DMBI, an n-type dopant, in a solution-processable method, and electrical conductivity was significantly increased up to ca. 0.19 S m^−1^ in 2010 [33]. Furthermore, fulleropyrrolidinium ions (FPI) and FPI-doped PCBM were produced to form film via a simple solution process by Li and co-workers, and its conductivity achieved 3.2 S m^−1^. The doping mechanism elucidated that electron transfer between the iodide on FPI and fullerene core could reach effective n-doping and high conductivity [34]. Although fullerene and its derivatives possess limited TE properties compared to other materials, they still have abundant potential to further improve their performance through molecular engineering.

#### 2.1.2. Quantum Dots

Quantum dots (QDs) have many things in common with real atoms, also knowns as ‘artificial atoms’, which were used to synthesize a novel lower dimension structure in the 1980s, and their properties can be adjusted more easily than atoms with requirements [36]. After more than 30 years of development since their discovery, with the in-depth research of quantum dots and the continuous improvement of preparation technology, quantum dots have been widely used in biology, electronic devices, and other fields [37,38,39].

**Figure 4:**
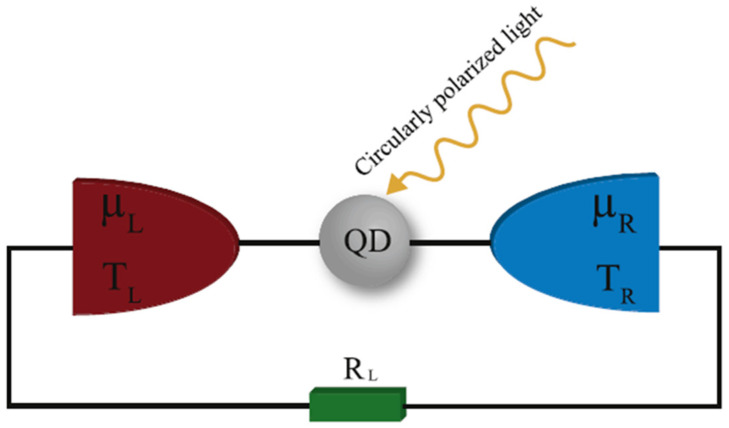
Schematic of a QD heat engine irradiated by a circular polarized light. Reproduced from ref. [41] with permission from Elsevier.

A significant application of QDs is the quantum dot TE engine. A new type of TE conversion device, the nano heat engine can break through the original constraints of traditional energy conversion devices and then change people’s habitual thinking mode of energy conversion, which expands a new space for energy conversion technology [40]. Liu et al. designed a QD heat engine (Figure 4a) to trigger a different voltage and a thermal bias. They proposed the relationship between parameters and the thermodynamic performance of the nano-TE devices. Additionally, the underlying mechanism of conversion performance of the nanoscale thermoelectric energy harvester was determined, which breaks the Wiedemann–Franz law without Coulomb interaction and induces spin-up and spin-down transport channels and the prohibition of double occupancy of electrons in the dot with Coulomb interaction [41]. Adam et al. studied heat-driven transport in serial double QD devices and disentangled the phonon-assisted transport effect and conventional thermoelectric transport effect, proving that phonon-assisted transport is sensitive to excited states [42]. These theoretical studies are not directly applied in this paper, but they might guide future research on high-performance TE devices.

**Figure 5:**
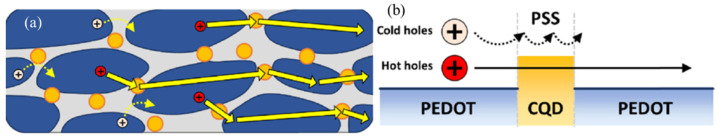
(**a**) Schematic of charge transport pathways of matrix which QD provide. (**b**) Schematic of PEDOT:PSS/CQD composites. Adapted from ref. [44] with permission. Copyright 2021 American Chemical Society.

Although most research on QD TE focuses on physical calculation, there are not many studies on composites. Unlike a quantum heat engine, QDs applied in composites interact with conductivity polymers or other materials, which always attain on the surface of polymers or others [43]. QDs provide added charge transport pathways across the insulating domains of the matrix and facilitate lattice hopping transport (Figure 5a). Due to the filtering effect, QDs are embedded and cold holes are filtered out, simultaneously improving carrier mobility and increasing *S* and *σ*. Moreover, based on this, Kilwon et al. also observed the reduction of *κ* in PEDOT:PSS/CQD composites (Figure 5b) [44]. Doping QD with other matrices is a fresh perspective for organic TEs and a strategy to enhance power factor with inevitably existing non-conducting domains in organic materials.

## 3. One-Dimensional (1D) TE Materials

### 3.1. One-Dimensional (1D) Materials

For TE devices, 1D nanostructures, such as superlattices, nanotubes, nanowires, etc., have demonstrated that could reinforce the TE composite’s performance [45,46,47]. Boundary scattering is enhanced, and *κ* could be reduced because 1D materials at the nanoscale are less than the mean free path of phonons. Meanwhile, *σ* could be raised due to retained and improved carrier transport in the 1D direction. For this reason, 1D structures are seen as the most promising materials in organic TE composites [48,49]. In this section, the recent progress of 1D materials will be presented.

#### 3.1.1. One-Dimensional Conductive Polymers and Related Composites

The quality of conductive polymers, as a matrix of TEs, largely determines the performance of their composites. One-dimensional conductive polymers possess relatively high conductivity, environmental stability, better dispersity, a short diffusion path, etc., which is hot research when applied to sensors, painting, and flexible devices [50,51]. This part will be unfolded based on different conductive polymers.

**Figure 6:**
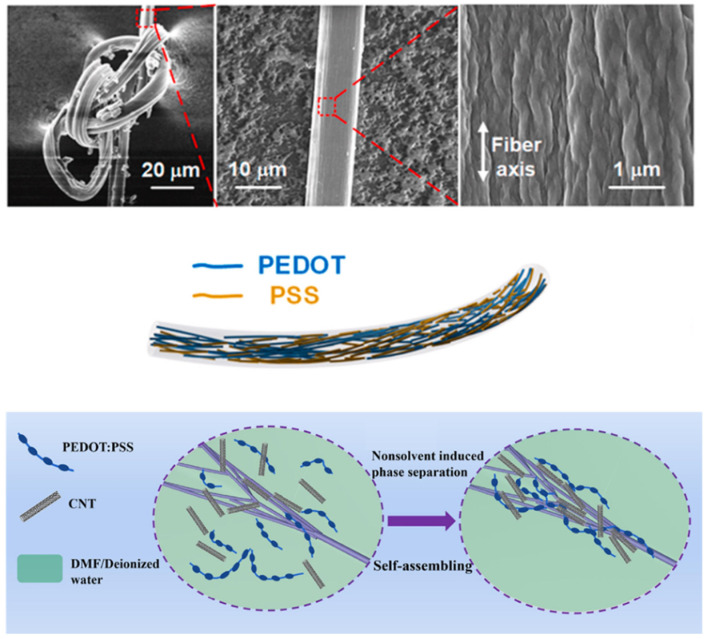
Scheme of some PEDOT fibers and composites. Adapted from refs. [50,58] with permission from Elsevier.

Poly(3,4-ethylenedioxythiophene) (PEDOT) and its composites, such as PEDOT:PSS, are among the best high-performance TE polymers, which exhibit higher electrical conductivity than other conductive polymers reported in the literature. Interestingly enough, PEDOT tends to display different TE performances with diverse nanostructures, generally following this order: nanofibers > nanotubes > nanorods > nanoparticles > bulks. As disclosed in Figure 6, 1D conductive polymers have a more orderly molecular chain, better crystallization, and higher carrier mobility [50,52,53,54,55]. Although 1D TE PEDOT has attracted interest from researchers, most of the fabricated PEDOT can only be called quasi-1D material because their scales are over 100 nm. However, the nanoscale always depends on synthetic methods, including hard template methods, soft template methods, interfacial polymerization, solution polymerization, and electrical polymerization. The size of conductive polymers fabricated by hard template methods usually is limited by models [56]. In soft template methods, the sizes are dominated by surfactant concentration and the categories of an inorganic acid. It is a fine way to synthesize a large proportion of conductive polymers. For instance, 1D polyaniline (PANI), one of the most popular nano-conductive polymers, can be synthesized by monomers combined with polyelectrolytes’ chains which have groups for forming hydrogen bonds and/or opposite charges, such as DNA, PAA, and P(VM-*co*-MA). In contrast to the frequently reported formation of 1D PANI, seldomly seen is the preparation of 1D PEDOT and its derivatives, which is caused by its dense growth to a compact morphology [57,58]. Therefore, it is necessary to develop methods for preparing excellent PEDOT and other conductive polymers for TE composites. Currently, the main way of making PEDOT fibers is wet-spinning. Doping concentrated sulfuric acid or DMSO, DMF, etc. Organic solvents would assist the transformation of aqueous PEDOT into high solid content. Further optimizing spinning conditions, coagulation baths, and post-treatment of PEDOT fibers are necessary to achieve high TE performance [59,60]. Chen et al. reported the preparation of PEDOT:PSS fibers with the highest electrical conductivity of 1013 ± 32 S cm^−1^, a simple and flexible TE generator fabricated using such fibers has stable output performance under environmental conditions [61].

As another widely used approach, electrochemical polymerization carries out oxidation and reduction reactions simultaneously on the anode and cathode, respectively. The polymerization processes can be controlled precisely by potential, solvent, ion, monomer structure, and so on. Moreover, the polymers obtained by electrochemical synthesis are not necessary to separate and purify due to the overall process without surfactants and/or oxidants. Furthermore, the composite’s doping level, morphology, and thickness of polymer film could also be turned into electrochemical processes. Therefore, numerous multifunctional 1D polymer matrix composites were fabricated by electropolymerization and templates. PANI and polypyrrole (PPy) composites can also achieve the same high TE performance as PEDOT composites with electrochemical polymerization. Additionally, PANI-based composites can be synthesized by electrochemical polymerization and electrochemical deposition. Recently we prepared PANI-based TE composites with a maximal PF of 236.4 ± 5.9 μW m^−1^ K^−2^ through dynamic three-phase interfacial electropolymerization of aniline in addition to the introduction of dimethyl sulfoxide (DMSO) and physical mixing with the single-walled nanotubes (SWCNTs) [62]. On the other hand, composites are able to be formed directly during the electropolymerization process. Huang et al. synthesized graphene/polyaniline composite film, which exhibited high conductivity with a one-step electrochemical process. Here, PANI architectures depend on graphene’s morphology; hence, customization of the PANI architecture would be realized by electrochemical polymerization [63].

**Figure 7:**
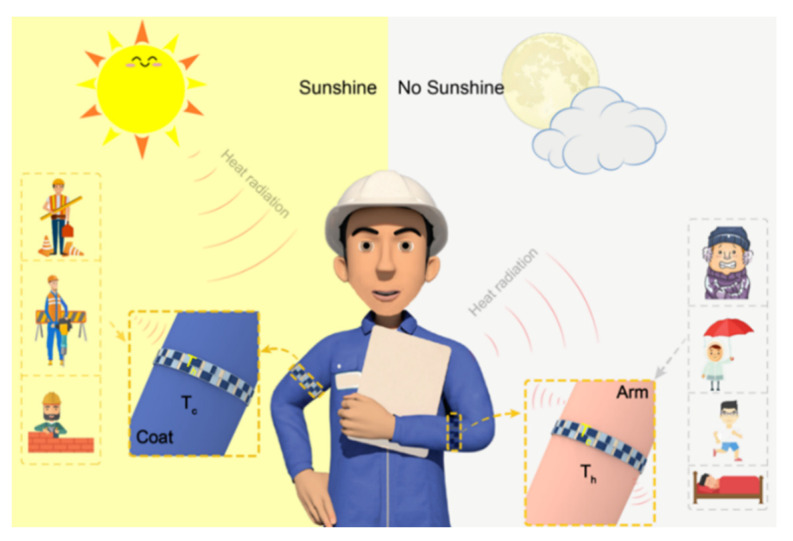
Scheme of smart, flexible photothermoelectric strip. Reprinted from ref. [65] with permission. Copyright 2020 American Chemical Society.

PPy not only possesses good conductivity but is also an outstanding photothermal agent, which has a wider heat resource than other conductive polymers. Thus PPy-based composites have enormous potential to generate intelligent devices [64]. However, only a few PPy-based composites combine photothermal with TE properties to fabricate multifunction devices. Lin et al. prepared a flexible photothermoelectric strip which contains coated fabric made up of PPy and PEDOT:Tos with Ag particles (Figure 7), outputting from 294.13 to 536.47 μV with the highest power density up to 13.76 nW m^−2^ [65].

#### 3.1.2. Carbon Nanotubes (CNTs) and Related TE Materials

**Figure 8:**
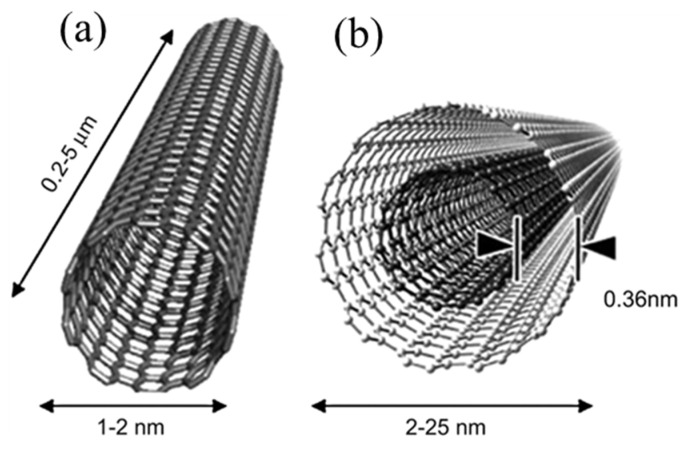
The schematic diagram of (**a**) single-walled carbon nanotubes (adapted from ref. [74]. with permission. Copyright 2002 WILEY-VCH Verlag GmbH, Weinheim, Fed. Rep. of Germany) and (**b**) multi-walled carbon nanotubes (adapted from ref. [75] with permission from Elsevier).

Carbon nanotubes, including single-walled carbon nanotubes (SWCNTs) with a diameter of 1–2 nm formed by curling a layer of graphene and multi-walled carbon nanotubes (MWCNTs) with a diameter range of several to hundreds of nanometers formed by curling multilayers of graphene, possess high conductivity, stretchability, and tenacity, demonstrating great potential as flexible TE materials confirmed by theoretical prediction and experimental results (Figure 8) [66,67]. Because of the unique structure of CNTs, they have obvious advantages in using charge transfer doping to adjust the charge carrier density, as well as excellent electrical and mechanical properties and large specific surface area, thus providing new ideas and directions for the development and preparation of high-performance flexible TE materials and devices [46,68,69]. In past decades, CNTs have evolved into the mainstream fillers in TE composites following these reasons: (1) high electrical conductivity to enhance TE efficiency; (2) large specific surface areas to form highly efficient interface; (3) ability to coat polymers on their surface to reduce thermal conductivity; (4) carbon-based TE composites with flexibility, environmental friendliness, biocompatibility, and low-cost [8,70,71,72]. However, the TE properties of CNTs-based composites depend on the CNT’s quality, so SWCNTs, which have all the properties that are better than those of multi-walled carbon nanotubes, are widely used to prepare TE devices [73]. Recently, Chen et al. developed an S-shape TE generator with flexible and foldable composite films. Electrical conductivity of PEDOT:PSS/SWCNT composites was improved from 1063 ± 80 to 1562 ± 170 S cm^−1^ after being post-treated by ionic liquid of bis(trifluoromethane)sulfonimide lithium salt (LiTFSI). The 30 wt % SWCNTs composites had the Seebeck coefficient of 21.9 μV K^−1^. SWCNTs play a pivotal role in the TEG fabricated using such materials in which strong interfacial interactions between components change the conformation of PEDOT chains [8].

**Figure 9:**
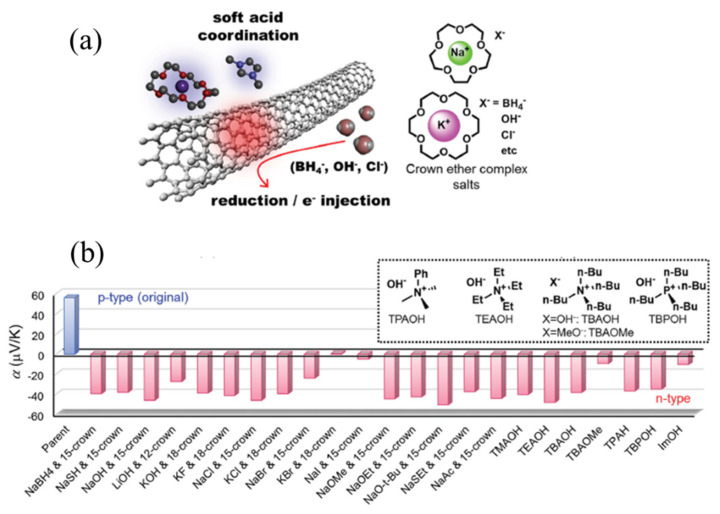
(**a**) Scheme of n-type doping with salts. (**b**) The Seebeck coefficient of 23 typical n-type inducers doping with SWCNTs. Adapted from ref. [79] with permission. Copyright 2016 WILEY-VCH Verlag GmbH & Co. KGaA, Weinheim, Germany.

High-performance organic TEGs require p-type and n-type elements to form a circuit in series. However, the development of n-type organic TE materials is relatively backward. Although the mobility of n-type organic semiconductors is reasonable, their intrinsic carrier concentration is mostly low, and their electron affinity is low, which is difficult to meet the requirements of TE conversion. In addition, the lack of high-performance materials also retards the progress of related theoretical research in this field. Therefore, to promote the development of the organic thermoelectric field, it is urgent to strengthen the development of high-performance n-type organic TE materials. For this plight, CNTs seem to be a great solution; however, CNTs and their composites are usually p-type in the ambient conditions without chemical treatment, n-type CNTs and composites doped with redox dopants are easily oxidized in the air, and the Seebeck coefficient becomes positive again gradually [76,77,78]. Against all odds, some progress was achieved through the unremitting efforts of researchers. Kawai et al. converted p-type SWCNTs to n-type SWCNTs in a creative way which utilized simple salts as dopants. SWCNTs treated with crown ethers complex salts and onium salts could present n-type TE performance, which showed air- and heat-stability at 150 °C (Figure 9a). As shown in Figure 9b, the Seebeck coefficient of 23 typical n-type inducers doping with SWCNTs varied with different salts and crowns, and this method is applicable in nanocarbon such as MWCNTs and graphene [79]. Although previous researchers have realized many achievements, there are still great efforts to be made.

## 4. Two-Dimensional (2D) TE Materials

### 4.1. Two-Dimensional Materials and Their TE Composites

The use of 2D materials is increasingly prevalent, particularly as a cornerstone in the construction of complex composite materials, due to their ultrathin thickness, large specific surface area, many active sites, short charge transfer distance to the surface, etc. A layered microstructural backbone can be constructed by the assembly of these 2D building blocks. The highly uniform and ordered channels formed could penetrate the whole backbone, which reinforces the TE conversion capacity in the vertical direction [80,81,82].

These typical 2D materials include graphene, black phosphorus (BP), transition metal dichalcogenides (TMDCs), MXenes, etc., which exist in different allotropes with preeminent electronic and optical properties. In a 2D structure, the Seebeck coefficient could be enhanced by the sharp features in the density of states (DOS) and intrinsic discontinuities, owing to quantum confinement. Moreover, the band gap of 2D materials is tunable, which could be achieved by transforming the number of layers, components of materials, and so on [83,84,85]. The following subsection will introduce several common 2D materials used in organic TE composites.

#### 4.1.1. Graphene

Recently, graphene has become increasingly popular in various fields due to its excellent electrical, mechanical, optical, and thermal properties. Two-dimensional graphene, one of the most conductive materials, is widely used for conductive polymer composites, which could enhance the conductivity of composites. [86,87,88] Regarding its application in TE materials, studies on graphene are usually divided into two categories. One is to investigate the thermal and electrical transport properties of graphene, especially the influence of graphene’s special edge carbon chain structure on phonon scattering and thermal conductivity regulation inside the material so as to optimize the TE properties of graphene [56,89,90,91]. However, research in this regard is a mostly theoretical calculation, and the actual application of graphene itself as a TE material is still a long way off [92,93,94,95]. Another kind is graphene composites with other materials in a proper way to form optimal TE materials [96,97]. Two-dimensional graphene’s lamellar structure and large specific surface area provide favorable conditions for the formation of nanocomposites with other TE materials. Meanwhile, the ultra-high carrier mobility of graphene is expected to improve the electrical properties of composite materials, while the low-dimensional nanostructure of graphene and its special boundary atom composition are conducive to enhancing phonon scattering and reducing the lattice thermal conductivity of composite materials to achieve collaborative regulation of material TE transport performance [98,99,100]. The following studies focus on the combination of graphene with conductive polymer for the formation of performance-optimized bulk or thin film TE composites.

Cai et al. fabricated polyaniline/graphene nanosheets (PANI/GNs) TE composite as pellets and films. Electrical conductivity and the Seebeck coefficient rose synchronously with an increased amount of graphene. Increasing carrier mobility seems to be a method with immense potential to make efficient composites in TE devices [101]. Although the power factor of the composite reported above cannot satisfy practical needs, it laid the foundation for future graphene composites. Chen et al. creatively designed a composite in which PEDOT:PSS was inserted into 2D graphene oxide (rGO/rPEDOT:PSS) layers. The rGO/rPEDOT:PSS exhibit high TE performance with excellent TE stability and mechanical flexibility for self-powered wearable devices. This wearable TE device could accurately recognize hand movements, including “Point”, “Pinch”, and “Grip” patterns, with a clever device design and optimization algorithm. The enhanced TE performance of composites is attributed to the sp^2^ carbon carrier channels in rGO, PEDOT:PSS carrier type, and regulated concentration [81].

At present, the theoretical research on the application of graphene in TE materials is mostly limited to monolayer or few-layer graphene, while the graphene used in experimental studies is mostly multilayer graphene or rGO, which is easily synthesized. In addition, graphene nanoscrolls, an emerging material, are similar to carbon nanotubes in structure but have a larger doping area than carbon nanotubes, which can introduce more charge carriers, thus improving electrical performance [102,103]. However, there are few reports on graphene nanoscrolls being used for thermoelectric composites for the time being, although it is promising in thermoelectrics. Therefore, the influence mechanism of multilayer structure graphene on electrical and phonon transport should be explored actively, and the possibility of improving the performance of composite TE materials is elucidated from the perspective of theoretical research. The novel monolayer graphene-based composite TE material and its synthesis and preparation technology should be developed simultaneously.

#### 4.1.2. Black Phosphorus (BP)

**Figure 10:**
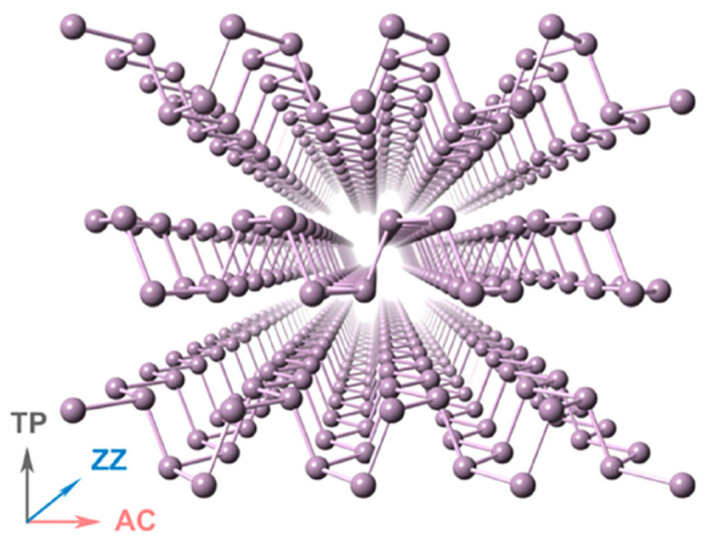
The lattice structure of phosphorene. Adapted from ref. [105] with permission from AIP Publishing.

Monolayer or few-layer BP, so-called phosphorene, was separated from bulk BP in 2014 as a promising 2D material in electricity and optics. After graphene, the distinctive structure and interesting anisotropic properties of BP, as another essential 2D material, triggered theoretical and experimental research in many fields. BP crystals were first discovered more than 100 years ago. It is the most stable among the allotropes of phosphorus (e.g., white, red, and violet phosphorus). BP crystals are wrinkled honeycomb layers that stack together by weak van der Waals interactions (Figure 10), which make it possible to prepare BP nanosheets by mechanical or ultrasonic exfoliation [104].

However, most of the research on black phosphorus in the field of thermoelectricity has focused on theoretical calculations. To a large extent, it is caused by the difficulties in obtaining monolayer black phosphorus and air sensitivity. Fortunately, monolayer BP has been obtained by some advanced methods of exfoliation, and appropriate dopants could improve BP’s air stability [106,107,108]. In one of the few experimental reports, Song et al. prepared PEDOT:PSS/BP composites and the TE properties of the films were considerably enhanced after the addition of BP, and the power factor could be increased by 109%. As the BP wt % increases, the mobility increases, and the carrier concentration decreases, which leads to an increase in both the Seebeck coefficient and the conductivity [109]. BP as a filler for TE composites is size-dependent, and the upper limit of the improvement of TE properties of high-quality 2D nanosheets is still unknown owing to the limitation of fabrication technology.

#### 4.1.3. Transition Metal Carbides/Nitrides (MXene)

MXene is a new frontier nanomaterial for flexible and stretchable electronic device fabrication due to its excellent electronic and metallic conductivity, rich surface functionality, and superior electrochemical and optoelectronic properties. Moreover, its tunable chemical surface makes MXene a surprising prospect for various applications. M_*n*+1_X*_n_*T*_x_* is the general structure formula of MXene, where *n* + 1 is the layers of early transition metals M, X is carbon and/or nitrogen, and T*_x_* stands for the functional groups on the surface of MXene. It should be noted that most MXene is metallic, but some MXene with M as Ti, Hf, Cr, Mo, Sr, Y, and W can exhibit semiconducting properties, so only these semiconducting MXene have some TE properties. In addition, due to the special structure of MXene, they are able to be made into n-type [93,110,111,112].

**Figure 11:**
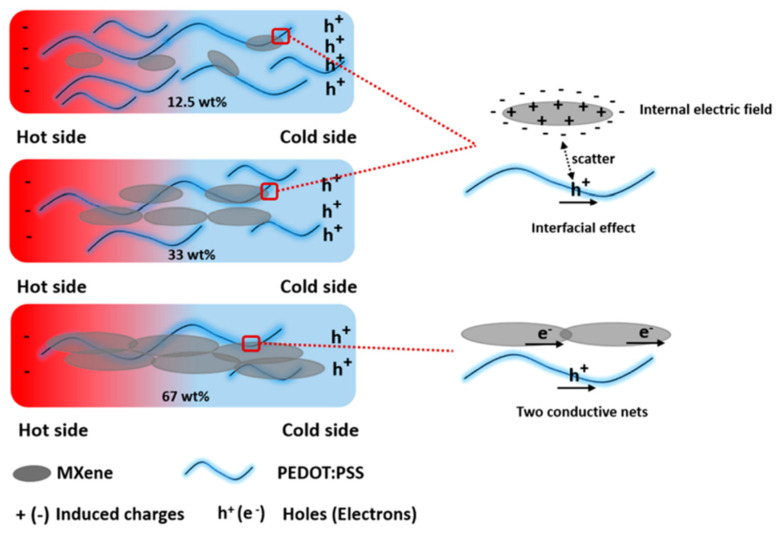
Schematic illustration of the MXene/PEDOT interfacial effect with loading MXene. Adapted from ref. [113] with permission. Copyright 2020, American Chemical Society.

Conductive polymers (such as PEDOT, PANI, and PPy) doping with MXene to fabricate TE composites is a new strategy. Ouyang et al. synthesized an n-type 2D MXene (Ti_3_C_2_T*_x_*), which can be blended into the p-type PEDOT:PSS. The Seebeck coefficient of PEDOT:PSS could be enhanced from 23 up to 57.3 μV K^−1^, and the power factor could be increased from 44.1 up to 155 μW m^−1^ K^−2^. The enhancement of the Seebeck coefficient is attributed to the energy filtering of the charge carriers by the internal electric field generated by the electron transfer from MXene to PEDOT:PSS. The internal electric field filters the low-energy charge carriers, thus improving the Seebeck coefficient (Figure 11) [113]. In particular, the method of preparation of MXene-based conductive polymer composites is one of the reasons that affect the performance of the composites. Popular methods include coating, vacuum-assisted filtration (VAF), in situ polymerization, and electrochemical deposition (ED). The choice needs to be determined depending on the characteristics of the matrix and filler [114].

## 5. Three-Dimensional (3D) TE Materials

### 5.1. Three-Dimensional Materials and Their TE Composites

Most of the low-dimensional materials mentioned above also have three-dimensional precursors. Optimizing the structure of these 3D materials to achieve the performance of low-dimensional materials is very attractive.

The TE performance of bulk materials is limited by the correlation between individual parameters, for example, the difficulty of reducing thermal conductivity while maintaining electrical conductivity [115]. However, these relationships can be decoupled, and their performance can be significantly enhanced through the rational design of nanoscale structures. The general strategy is to add nanostructures, such as nanoarray, random pores, and uniform patterning, to the bulk material to improve the TE properties, the reduction of thermal conductivity resulting from phonon scattering and the enhancement of the Seebeck coefficient arising from energy filtering. Unfortunately, these approaches have limitations in their applications. The high performance exhibited by nanoarrays depends on the high quality of 0D and 1D materials, and scaling up production is challenging. In contrast, random porous structures are easier to obtain, but the morphology of these nanostructures is uncontrollable and therefore has limited improvement in TE performance [116,117,118]. The lithographic process is a method to achieve high porosity in a controlled manner, but it is expensive and only suitable for preparing precision devices [119].

#### Metal-Organic Frameworks (MOFs)

MOF is a multi-porous coordination polymer composed of inorganic metal nodes and organic linkers, which has become a promising multifunctional material due to its high porosity and highly tunable composition/structure and has been investigated in various applications such as gas storage, separation, and catalysis. The high porosity of MOFs offers a new strategy to improve TE properties since the pores can strongly scatter phonons, thus reducing thermal conductivity and increasing TE properties. Although most MOFs are insulators, their electronic structure can be tuned so as to promote electron flow. The *ZT* values of current MOF-based TE materials are not satisfactory, but the unique structure of MOF gives it enormous possibilities for optimization, especially for n-type materials [72,120,121].

## 6. Summary and Outlook

Although composites consisting of conductive polymers and fillers of various architectures have made significant progress in TE applications, they still lag behind inorganic TE materials which have a maximum *ZT* value of over 2. An efficient optimization strategy is to make full use of the quantum effect to enhance the TE performance of the composite, which can be realized via the selection of a suitable filler that matches the architecture of conductive polymers [70,122].

In general, the low dimensionality of composite architecture can greatly improve TE performance. This mini review summarizes and discusses the signs of progress made in recent years in TE materials containing fillers of different dimensions. Concluding the characteristics of the above materials, the following ideas are provided to shed light on the development of novel high-performance TE composites based on conducting polymers. 0D materials, such as fullerene and QDs, should be further taken into account to modify surface groups as dopants to increase composites carrier mobility or/and phonon scattering. Although 1D materials have proved to be the ideal TE material, continuing optimization of their preparation methods and structure regulation is still on the way to enable further improvement of their TE performance. For 2D materials, changing interlayer actions is able to enhance TE performance. Three-dimensional materials play a key role in the fabrication of precision devices using organic TE composites. Perfect matching of 0D materials with 2D and even 3D matrices may be an effective way to improve the TE performance of composites. The precise design and realization of the structure and morphology of conductive polymers are believed to significantly influence interactions among constituents of composites consisting of polymers and fillers with varying architectures and dimensions, thus optimizing TE performance of the composites and TE devices fabricated thereof. Theoretical studies such as DFT calculation are useful tools in solving intricate interrelationships between materials structure and its TE performance to a certain extent. As long away as it is, we believe the strenuous efforts of researchers in the fields of materials, energy, and electronics will accelerate the progress in the commercialization of conductive polymer-based TE composites and TE devices of different functions. We hope this mini review is helpful to researchers in the application of TE materials by providing further understanding of the essence of materials of different dimensions and the interplay between their structure and TE properties in light of current advances in related fields.

## Data Availability

Not applicable.

## Sample Availability

Samples of the compounds are not available from the authors.
